# Sensoring a Generative System to Create User-Controlled Melodies

**DOI:** 10.3390/s18103201

**Published:** 2018-09-21

**Authors:** María Navarro-Cáceres, Wataru Hashimoto, Sara Rodríguez-González, Belén Pérez-Lancho, Juan Manuel Corchado

**Affiliations:** 1 BISITE Research Group, University of Salamanca, Calle Espejo, S/N, 37007 Salamanca, Spain; srg@usal.es (S.R.-G.); lancho@usal.es (B.P.-L.); corchado@usal.es (J.M.C.); 2 Department of Computer Sciences, Osaka Institute of Technology, Kitayama 1-79-1, Hirakata 573-0196, Japan; wataru.hashimoto@oit.ac.jp

**Keywords:** mechanical device, hand movements, CBR, melody generation

## Abstract

The automatic generation of music is an emergent field of research that has attracted the attention of countless researchers. As a result, there is a broad spectrum of state of the art research in this field. Many systems have been designed to facilitate collaboration between humans and machines in the generation of valuable music. This research proposes an intelligent system that generates melodies under the supervision of a user, who guides the process through a mechanical device. The mechanical device is able to capture the movements of the user and translate them into a melody. The system is based on a Case-Based Reasoning (CBR) architecture, enabling it to learn from previous compositions and to improve its performance over time. The user uses a device that allows them to adapt the composition to their preferences by adjusting the pace of a melody to a specific context or generating more serious or acute notes. Additionally, the device can automatically resist some of the user’s movements, this way the user learns how they can create a good melody. Several experiments were conducted to analyze the quality of the system and the melodies it generates. According to the users’ validation, the proposed system can generate music that follows a concrete style. Most of them also believed that the partial control of the device was essential for the quality of the generated music.

## 1. Introduction

Artificial Intelligence gave solutions to many problems encountered in Biology, History, Mathematics and other fields. The rapid evolution of Artificial Intelligence in recent years, has attracted the interest of researchers in Computer Sciences and Arts to the field of Artificial Creativity. This discipline uses technology to imitate the human creative process with the aim of generating works of art [1], state of the art proposals are concerned with several artistic fields, such as visual arts [2,3], narrative [4,5] and music [6,7].

Many different techniques have been applied to generate music automatically. Evolutionary computing comprises a variety of algorithms that follow the natural behavior of algorithms and they have been applied in numerous fields of research to solve problems. This approach is widely used to generate interesting musical products. Ref. [8] proposed a chord sequencer based on evolutionary methodologies. Ref. [9] presented an accompaniment system based on emotions by applying a genetic algorithm. Ref. [7] analyzed different evolutionary methods for generating valuable music.

Statistical methods are also commonly used in the generation of music. The deep analysis of different statistical models performed in [10] is particularly interesting. We should also remark that Markov Models are used very commonly to generate different kinds of music [6,11,12].

More recent works are focused on the collaboration of humans and machines for the generation of valuable music. Usually, human-machine interaction is established through mechanical devices which capture the intentions of the users. For instance, in [13] a device was used to compose music from the movements of people with disabilities. In [14] touchscreens were used to generate music. Ref. [14] proposed an interactive framework that allows musicians to compose music through hand motion and gestures.

Some research works incorporate the movements captured by the devices to create a learning tool that generates new music. Flow Machines [11] is an illustrative project which proposed the creation of music through collaboration with human musicians, who can lead the creative process using an MIDI instrument. Likewise, VirtualBand [15] generated jazz compositions following the performance of a melodic instrument, which is captured through a sound card and a recorder. GimmeDaBlues [16] automatically generated the bass and drums parts while the user played on keyboard and/or solo instruments, responding to the user’s activity.

The majority of previous proposals require users to have some musical knowledge, especially when musical instruments are involved. This work aims to overcome this limitation by designing an intelligent system which will allow users with no musical training to generate music. The design includes a mechanical device that looks like a joystick, the user moves it to create a melody.

The system stores the different user profiles in order to adapt the composition to each specific user and allows them to select different musical styles based on different musical contexts and prominent composers in History. Two execution modes are also presented: a supervised mode and a collaborative mode. The first one aims to learn from the indications given by the user through the mechanical device. In the second mode, the machine guides the user through the automatic movements of the device, giving the user the opportunity to learn how to generate a good melody according to their preferences and the style. In both cases, the goal is to obtain a creative product from the communication between humans and machines. The involvement of the mechanical device is essential for the purposes of this work because, on the one hand, the machine learns from the users’ indications through the mechanical device, and on the other hand the user can learn about composing music in a specific style when the machine guides the movements of the user through the device.

In this work, it is not necessary for the user to have musical knowledge. We have established a creative collaborative process, between an AI model, which learns to compose music following a certain musical style, and a user who directs and supervises the composition by interacting with an intuitive device. The collaboration allows the final melody to be adapted to the taste of the user or the context, that is, a certain situation, a narration or a sequence of images, just to name a few examples. Future developments of this system could be useful for the creation of short soundtracks, or as a tool for psychological or sociological studies that seek to explore the descriptive capacity of music, its influence on the behavior of people, or even the possibilities it offers as a means of communication or therapy for people with problems such as autism, dementia, brain injuries, etc.

We also point out that MultiAgent Systems (MAS) have been used in proposals as an efficient tool for solving problems in a variety of contexts. An agent is any entity that captures process output data in the context to perform different actions to change something in the environment. The organization of agents permits to improve flexibility and efficiency in solving problems in different and dynamic environments. In music, they are successfully applied and generate very interesting results in terms of creativity [17,18].

Encouraged by the successful performance of agents in creative fields, we present a MAS that governs our system. The MAS provides the system with scalability and flexibility, , which permit to make changes in problem specification, such as changing the composition algorithm or user preferences, without changing the structural composition. To improve users’ experience, the system must learn from their preferences. This is done by integrating a mechanical device in the system which is controlled by the user to influence the tonal and rhythmic aspects of a melody. Consequently, the MAS provides an agent with a Case Based Reasoning (CBR) architecture. This agent includes a training mechanism for the generation of different styles of music by making use of a memory of the previous melodies generated by the user and Musical Interface Digital Instrument (MIDI) files retrieved from the Internet. The CBR agent is provided with a Markov Model that is trained to extract the most likely sequence of notes according to the movements of the mechanical device and the retrieved MIDI files. The musical result is rated by the user in terms of its quality. The created melody may be stored in the memory for future use.

This article details the entire research process. The next section introduces some recent research related to multiagent systems (MAS). Section 2 describes the MAS architecture, the employed mechanical device and the overall workflow. Section 3 describes the learning core of the system, including all the details of the CBR agent. Section 4 details the experiments and provides a preliminary discussion of the results. Finally, Section 5 collects the main conclusions and suggests possible lines of work for the future.

## 2. Multiagent Systems and Music Generation

The concept of agents is present in various fields of study, such as psychology, Computer Science, Sociology, Medicine, Economics, etc., with different points of view and behaviors. In Computer Sciences, the term “agent” is widely used in very different research contexts, such as distributed artificial intelligence or human-computer interaction [19].

The implementation of multi-agent systems is an ideal option if we wish to create and develop open and heterogeneous systems, which are normally used in the composition process. The use of MAS facilitates the incorporation of new composition techniques in the system based on human-machine collaboration. Agents and creativity are two disciplines that have interacted in several cases studies before. Ref. [17] presents a toolkit for the design of interactive musical agents. The implementation of agents has allowed to identify issues that will be addressed in an upcoming study of user-centered design. Ref. [20] proposed SC-EUNE, a system that generated creative content based on the agent’s curiosity. Thanks to the agent’s architecture, curiosity and surprise behavior was easily implemented, and the interaction between entities was successfully developed within the system. Ref. [18] developed adaptive critics for evolutionary artists. The theoretical definition of any agent system’s behavior and message sharing, were key to developing behavior based on criticism. A creative agents approach, called motivational agents, was proposed and used to explore unknown environments [21]. Again, the general architecture of an agent was essential in this approach for creating behavior that developed motivation based on information sharing. Ref. [22] proposed Inmamusys, based on agents and expert systems for the generation of creative musical content. In this case, agents are essential for combining basic knowledge about music and the knowledge provided by expert systems. Ref. [23] proposed a MAS that does artistic musical performances. In all the mentioned works, the agents’ behavior allows to update the musical information through message sharing. Additionally, these agents could be changed very easily to slightly modify their behavior if necessary, which implies higher efficiency and modularity.

Interaction between computers and humans also plays an important role in the generation of music and this can be easily modeled with a MAS [24]. For example, ref. [25] described the Musebot and the Musebot ensemble to explore collaborative methodologies for the creation of collective compositions based on a combination of agents and communities. In this case, the agent is an entity that interacts with the user transparently, making the communication process easier for the users. Ref. [26] proposed a multi-agent system which generated melody pitch sequences. The agents have melodic intelligence and generate pitches as a result of artificial emotional influence and communication between agents, and the melody’s hierarchical structure is a result of the emerging social agent structure. Again, the agents interact with the users, and the results show the users are satisfied with this process, partly due to this encapsulation. This kind of behavior would be harder to model without the involvement of agents.

Therefore, the state of the art conveys the suitability of the MAS technology for modeling a system that generates music. In the present system, human-machine collaboration is essential. In the works described above, the interaction was encapsulated in one agent, which proved to be helpful in interacting with the user transparently. Additionally, MAS provides the system with flexibility which allows it to operate in an environment with dynamic elements such as melodic changes and user preferences.

Finally, the use of agents in the development of this system implies computational advantages in message exchange and behavior implementation . On the one hand, the use of the messages permits to separate the different tasks so that they work completely independently of each other, and to have better control of the access to resources. Additionally, the use of a MAS architecture makes it easy to change the behavior of the agents in the future. This can be done by improving old agents and adding new behaviors represented by new agents, without affecting the general system or the general architecture.

## 3. Related Work in the Field of Music Generation

Artificial Intelligence has been widely applied to problems in very different contexts, such as Biology, History or Mathematics. With the recent evolution of Artificial Intelligence, the field of Computational Creativity has attracted the interest of researchers in Computer Sciences and Arts. Therefore, Computational Creativity (CC) can be considered as an emerging branch of artificial intelligence (AI) that studies and exploits the potential of computers to be more than feature-rich tools, and to act as autonomous creators and co-creators in their own right. Proposals are made in different artistic fields, such as visual arts [2,3], narrative [4,5] and music [6,7].

The collaboration between humans and machines in the generation of valuable music is a current research trend within the CC. In the collaboration process, different sensors and devices are usually used to capture the users’ intentions and reflect them in the composition. For instance, Ref. [27] generated three melodies simultaneously, which are controlled by a wheel spun by 2 hamsters. In this case, the collaboration is established between an animal and the machine through the acceleration of the wheels, but the system does not consider any conscious preferences to be applied to an external context. Additionally, the preferences are not captured in anyway by the mechanical devices, as the hamsters do not decide on the composition of the melody. Inmamusys [22] presented a framework that allowed the user to pre-adjust some parameters for the composition of music. The system is based on evolutionary algorithms instead of statistical models, and the interaction with the system works as a setting panel, real-time collaboration is not allowed. Vox Populi [28] enabled users to interact with the system, which contained an evolutionary algorithm that composes music through different interactive parameters. The users could participate through the use of touchscreens and standard devices like mouse and keyboard. However, this approach is hard to understand for users without any previous knowledge of the system and without a previous musical training, as they can modify several musical properties at the same time through different interactive items. GimmeDaBlues [29] automatically generated the bass and drum components by learning from a user playing the keyboard and/or other solo instrument, thus it also is created for people with some musical training.

Statistical models are commonly used in the development of systems that generate music. In particular, Markov Models are widely applied as a generative algorithm that reproduces a particular style but creates original melodies. On the contrary, bioinspired algorithms or grammars usually need a predefined function or rules. There are a variety of works that, apart from the involvement of devices, also make use of Markov Models to construct a system that learns from the users. Ref. [13] use a device for composing music from the movements of people with disabilities. The work uses hardware sensoring to detect the movements of disabled people. Instead of generating different styles of music, the system uses these movements to create dance music. Flow Machines [11] provided new ways for creating music by collaborating with human musicians, but also learning from an existing corpus. Flow Machines always involve a musician who plays an MIDI instrument connected to the system. Thus, a user without specific musical training cannot interact with the system. Likewise, VirtualBand [15] generated jazz compositions following the performance of a melodic instrument. They use a learning model such as a Markov Model, but again a musician is required to collaborate in the process. The Continuator [12] composed music from a brief introduction that the musician makes with a musical instrument to provide some guidelines. Although it uses an advanced learning model based on Markov Models, The Continuator needs a musician playing an instrument to interact with the system.

The described works highlight that, although Markov chains can represent musical features successfully, they are limited by the musical properties related to the time, such as the phrases, the harmony or the hierarchical structure. That means Markov Models are indicated to generate short pieces of music with very good results, according to previous works, but they are not able to generate longer melodies with equal quality. By combining Markov models with human interaction we overcome the limitations of the Markov Models, as the responsibility for organizing the piece and organizing the structure is left to the user, who guides the generation of music through the device. Therefore, the advantages of Markov Models are fully exploited in the system, as they are only responsible to generate music according to a major plan given by the user.

Additionally, unlike previous proposals, this work aims to offer a tool for a potential user which can be (but not limited to) people without any musical training, considering it as a person who wants to create music for a specific purpose (ambient music, soundtrack, psychological or sociological study, music therapy, etc.) without knowledge of composition. The interaction has therefore been conceived through a device that allows intuitive movements to guide the composition giving it a certain character through basic actions on the melody (speed of the notes, more or less acute tone, intervals, inclusion of punctual rests, etc.) that can influence or provoke certain feelings in the people who listen to it. It could also be used as a tool for people with communication problems (children, the elderly, people with autism or brain injuries) in which music, exploration and play are a very interesting way of cognitive development or therapy.

## 4. Model Overview

**Figure 1:**
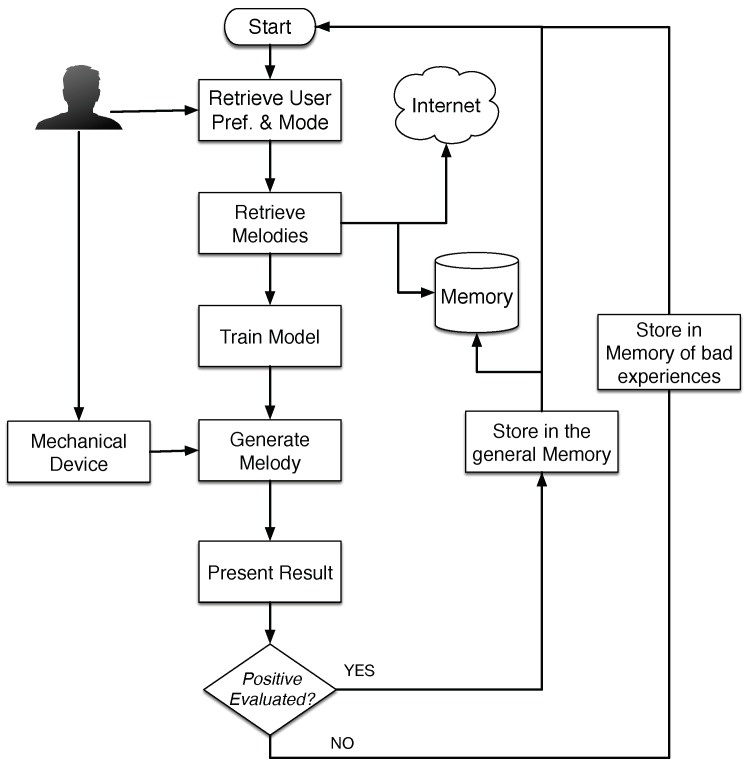
Illustration of the flow followed by the whole system.

In this section, we detail the overall structure of the developed system. One of the goals is to promote collaboration between humans and machines, in order to generate melodies according to users’ preferences, or context in which the system is used. Therefore, the system is equipped with a mechanical device that communicates human indications to the machine. The device consists of a mechanical arm that the user can guide to partially control the melody. Additionally, the machine learns from the users’ behavior in order to improve its final results. The system can work in different modes which will be explained in the next sections. Independently of the mode, the system follows the flowchart shown in Figure 1 that is implemented by the MAS architecture.

The figure represents the behavior of the main system interacting with the device. Initially, the user creates a profile and logs into the system. The profile contains information about melodies that have previously been generated and the user’s ratings. In the first step, the user has to select some formal parameters to generate the melody; they are related to the musical style, composer or tonality. With these options, the system searches for previous solutions that suit the description in the internal memory. If the system does not find enough solutions, it can retrieve external files from the Internet that comply with the parameters specified by the user; style, composer and tone. This information is used as a training set to calculate the probability of a note being selected. This probability is a combination between the statistical model that learns from previous experiences and through the indications of the user given by means of the mechanical device. Once the user has finished their melody, the whole result can be played again and should be evaluated. With the rating obtained from the user’s, the system determines whether the melody is good enough to be stored in the memory and used to train the Markov Models, otherwise it is discarded.

The next subsections describe the developed MAS and focus on the technical details of the mechanical device.

### 4.1. Multiagent Architecture

Among the different tools that were available for the development of our system, we selected multiagent systems, which provide certain advantages. A MAS can fulfill different roles that can be implemented by agents which behave differently, depending on the context. This feature provides the system with great flexibility, where the change of requirements influences agent behavior. MAS can also provide a global view of the problem and give possible solutions.

In order to design the agents that comprise the MAS, we analyze the needs and expectations of the system. The needs are fulfilled by the behavior of the different entities involved in the system. In our case:

- Interface Agent: This agent communicates the external user with the machine in order to collect their preferences and provide the final results.
- Device Agent: This agent translates the movements of the mechanical device into data that the system should evaluate in order to make decisions.
- Crawler Agent: This agent searches for new files on the Internet that fulfill the requirements of the users.
- Storage Agent: It is responsible for the storage and retrieval of the data saved in the memory.
- Composer Agent: This agent contains a CBR architecture and aims to generate a new melody based on previous experiences and the data given by the Device Agent.

**Figure 2:**
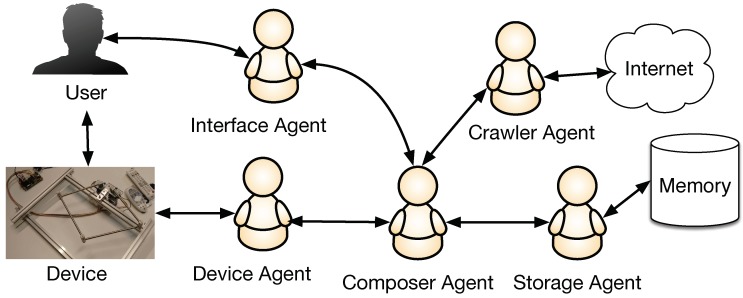
Illustration of the MAS developed for the system.

The Composer Agent is the main core of our MAS, implemented with a CBR architecture because this structure is perfectly suited to the requirements of the system. The communication of this agent with the rest of the MAS is shown in Figure 2.

As we described in Section 3, each user has a profile with past experiences and their preferences. Initially, the user selects the musical style and/or the style of a composer in order to generate a melody on its basis. Additionally, a tonality might be selected beforehand, if the user wishes to do so. The whole process is monitored by the Interface Agent, who also sends information to the Composer Agent. This agent makes use of the Crawler and Storage Agents to retrieve previous solutions or new musical files that fulfill the requirements stated by the users. In the learning core of the Composer Agent, a Markov Model is designed and trained with previously retrieved solutions. The Composer Agent then determines the new notes of the melody, which will depend on the mode (collaborative or supervised mode), the information of the movements of the mechanical device given through the Device Agent and the probabilities obtained with the Markov Model. Finally, the Composer Agent shows the results through the Interface Agent and receives a rating from the user. With this new melody, the Agent decides whether the melody should be stored by the Storage Agent, or whether it should be removed. The Composer Agent will be described in more detail in the next sections.

### 4.2. The Device

**Figure 3:**
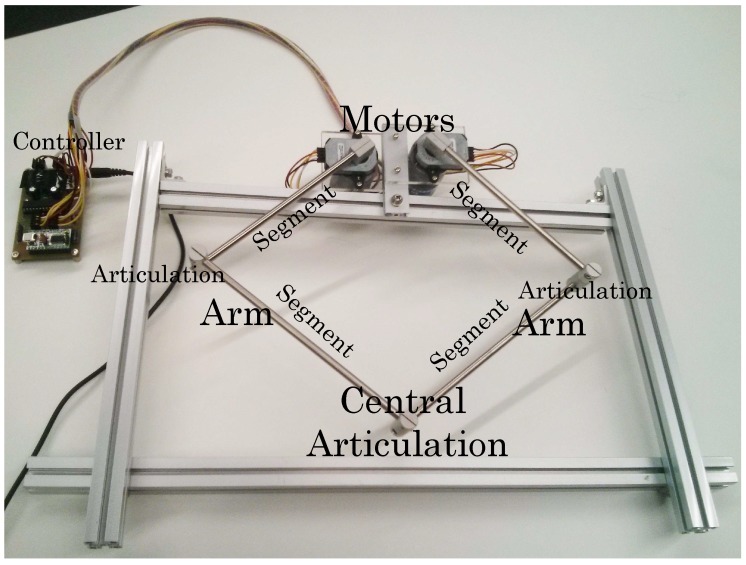
Illustration of the mechanical device.

Before going into details on the algorithm for the generation and control of melodies, we should first explain that the device used (Figure 3), has been partially developed by Professor Wataru Hashimoto from the ILO (Osaka Institute of Technology) [30]. The device consists of 2 interconnected motors anchored to an aluminum frame, to create a pantograph with 2 degrees of freedom. We also have two articulated arms with two segments (*L* and *l*) each, which are connected by a central articulation.

**Figure 4:**
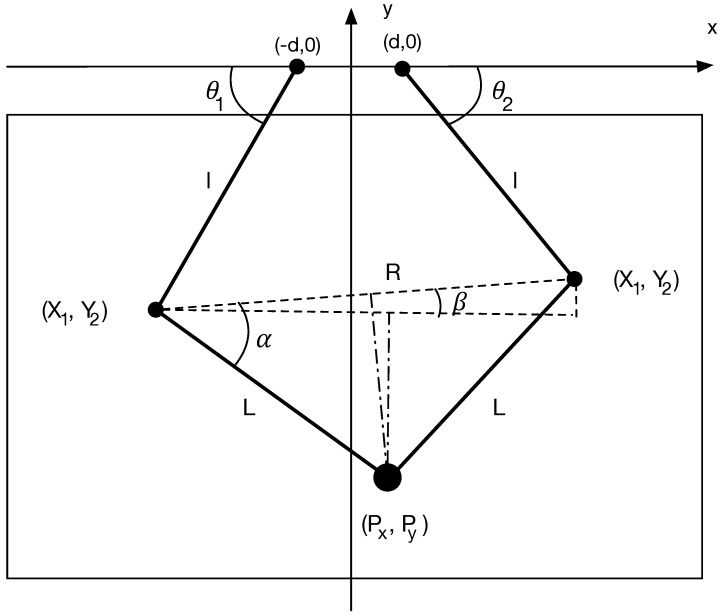
Representation of the coordinates and angles existing in the device.

Technically, the device contains a controller chip that uses a serial connection via a USB to RS232 adapter to communicate with the computer. The device buffer consists of 4 bytes, of which the first 2 correspond to the left motor and the last 2 to the right motor. Each motor sends the corresponding arm output angle $\theta_{1}$ and $\theta_{2}$ (Figure 4), with a value between 0 and 4096, and receives a value between −20,000 and 20,000 indicating the exerted torque. We can communicate with the device every millisecond through a constant read/write process.

The Device Agent makes all the pertinent calculations each time the position of the central articulation changes. In particular, the agent makes use of the values given by the mechanical device so that each pair of input angles $\theta_{1}$, $\theta_{2}$ of the motors could be translated into a particular position $P=(P_{x},P_{y})$ in the plane, as shown in Figure 4. Therefore, a system of trigonometric equations is applied, following the set of triangles generated by the distance *d* from the center of the motors to the segments *L*, *l* and the angles α and β. In particular, *P* is calculated as:

(1) $$P=\left(P_{x},P_{y}\right)=\left(X_{1}+L\cdot cos\left(\alpha -\beta \right),Y_{1}+L\cdot sin\left(\alpha -\beta \right)\right)$$

where $\left(X_{i},Y_{i}\right)$ are the coordinates of the articulation point of the arm, α is the angle between the line *R* that links $\left(X_{1},Y_{1}\right)$ with $\left(X_{2},Y_{2}\right)$ and β is the angle between the horizontal axis drawn from $\left(X_{1},Y_{1}\right)$ and *R*.

$\left(X_{i},Y_{i}\right)$ are calculated through trigonometric relationships according to Equation (2).

(2) $$\left(X_{i},Y_{i}\right)={\left(-1\right)}^{i}\left(d+l\cdot cos\theta_{i},-l\cdot sin\theta_{i}\right)$$

where $\theta_{i}$ are the angles between the segments and the horizontal axis, and *l* is the length of the lower *i*-segment. With these values, *R* can be easily calculated as the Euclidean distance of $P_{1}$ and $P_{2}$:

(3) $$R=\sqrt[{}]{{\left(X_{1}-X_{2}\right)}^{2}+{\left(Y_{1}-Y_{2}\right)}^{2}}$$

*R* allows to determine $\alpha =cos^{-1}\left(\frac{R}{2L}\right)$ and $\beta =tan^{-1}\left(\frac{Y_{1}-Y_{2}}{X_{1}-X_{2}}\right)$, which in turn allows to calculate the point *P* (Equation (1)).

One of the advantages of using this device is that the motors can exert a force of feedback that can be useful for the user in diverse applications, as in the case of this proposal, to assist the user in the collaborative mode. To apply a feedback force, the Device Agent determines the angle values that are sent to the motors to achieve the desired position *P* of the central articulation of the device following the calculi proposed before.

#### Controlling the Position

The essential role of the Device Agent is to translate the position of the central articulation into a note and a duration that could be called a “reference” note and duration. As the position *P* is mapped on a 2 dimensional space, it has been decided that the Y axis represents the reference pitch, since “climbing” is intuitively associated with higher notes and “lowering” with deeper notes. In turn, the X axis corresponds to the reference duration (rhythm). Silences are also controlled by the X and Y axes.

To ensure that all the reference points can be accessed through the use of the device, the coordinate space of the device has to be delimited. On the X axis, 0 represents the shortest note (sixteenth note) and 1 represents the longest note (round). On the Y axis, 0 represents silence and 1 represents the highest note in a high octave. Although the points along the Y axis are initially framed into three octaves, the octaves can be changed when the user pushes the device to its maximum *y*-position (to go to upper octaves) or minimum *y*-position (to go to lower octaves or silences).

Another peculiarity has been added to the control: the possibility of shortening notes which are too long from the user’s point of view. If a long note has been generated and the device position is driven to the left on the X-axis (towards 0, that is, towards shorter notes), at the time when the duration indicated by the reference is less than the duration elapsed from the beginning of the note, the current note is “cut” and the duration of the next one is then calculated.

## 5. The Learning Core: CBR Agent

**Figure 5:**
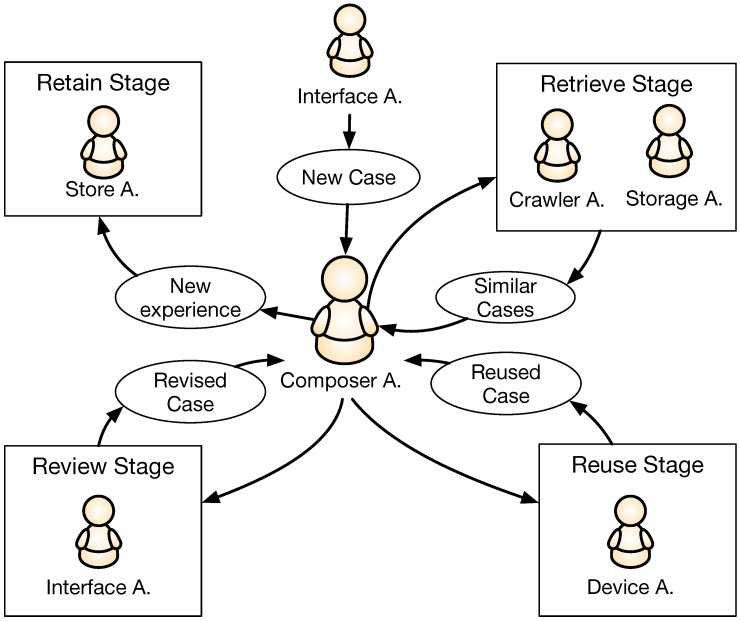
Schema with the CBR process. The case represents a formalization of a melody with the initial requirements submitted by the user, and the final review obtained.

As we explained in the previous section, the Interface Agent sends all the information on user preferences to the Composer Agent. These preferences are encoded as the initial labels that describe the melody the user desires to compose. The labels are text words which are related to the musical style or a specific author. Then, the agent starts with its cycle. Firstly, the previously generated melodies are taken from the memory of the system in the Retrieval Stage, thanks to the Storage Agent. When the Storage Agent has not stored enough solutions, the Composer Agent communicates with the Crawler Agent to retrieve external solutions from the Internet, that satisfy the initial parameters of the author and style. These solutions are then used to train a Markov Model that will be applied in the Reuse Stage together with the indications collected by the Device Agent to generate a new melody. The particular combination of the statistical model and the mechanical device depend on the working mode of the previously selected system. Two different modes are developed, namely, supervised mode and collaborative mode. The supervised mode gives the user total control of the mechanical device without any external assistance from the machine. On the other hand, in the collaborative mode the user can experience a resistance force in the mechanical device when the machine wants to influence the melodic result. Finally, the complete melody is rated by the user in the Review Stage through the Interface Agent to determine whether it should be stored by the Storage Agent to help improve the user’s experience, or discarded due to its low quality (always according to user preferences) in the Retain Stage. Figure 5 illustrates the main process of the CBR cycle.

The entire process is based on the concept of case, which can be defined as a 3-tuple *C* = <P,S,R>. *P* is the problem domain that represents the information of the composition we want to create, this includes the style, the tonality and the author, all encoded as labels. *S* is the sequence of <N,D> where *N* is the considered musical note and *D* corresponds to its duration, encoded in a standard format. Finally, *R* represents the users’ subjective rating about the obtained musical melody.

The first step is to obtain and process the input data detailed in Section 5.1. The process of generating the melody from the Markov model and the position of the device, as explained further on. Finally, Section 5.3 and Section 5.4 explain the process of feedback of the results.

### 5.1. Searching for Previous Solutions (Retrieve Stage)

Every past experience is stored as a 3-tuple *C* = <P,S,R>, and represents a previous case saved in the memory of the system. Each time the Composer Agent needs to generate a melody, a set of previous cases with similar properties is retrieved from the memory. In order to measure similarity between the past experience and the present one, the Storage Agent compares the *P* component with the input description.

In order to avoid difficult translations when encoding the case, the MIDI format (Musical Instrument Digital Interface, [31]) is used to encode the musical parameters, whose structure is completely standardized to permit an easy access to the information about pitches and durations. Additionally, MIDI files can be easily found on the Internet and are quite light, as they do not contain the acoustic waves, but instructions to reconstruct the song with a musical synthesizer.

The problem *P* is represented as a set of labels $L_{i}$. Each label $L_{i}$ represents the name of a musical style, such as classical, pop, rock, jazz, etc. and/or the specific composer of the music (i.e., Bach, Mozart, The Beatles, etc.). Sometimes if the music file gives that information, there is a label which describes the main tonality of the composition. The labels are extracted from the information of the MIDI files, which always contain some metadata labels related to the author, style and sometimes the key signature, and can be easily read. With this description, a comparison of the labels is made by the Storage Agent to retrieve the similar cases *C* to solve the present problem.

The number of melodies used for the future training of the Markov Model was empirically set to 100. It might happen that the number of solutions is not enough due to the introduction of new musical styles or composers. In such cases, the Composer Agent needs to collect more solutions with features that coincide with the user’s set of preferences. Thus, the Crawler Agent is required to search and retrieve labeled musical files that are available on different websites in order to generate a database with files labelled $L_{i}$. Although this is not the focus of the present paper, we give some details about the crawling process.

Most websites that offer large numbers of MIDI files for free usually have simple but varied structures. For example, at http://www.midi.com.au/ the structure could allow us to determine the style of each MIDI, but at http://www.download-midi.com/ (which also seems to be structured in a similar way) the download links are not structured by styles. Therefore, a general crawling strategy has been chosen to allow MIDIs to be downloaded from any page regardless of their structure: all the links found on an objective page are searched for MIDI files, and then all the links found on those pages (except those that have already been traveled), and so on until reaching a certain depth. Each time an MIDI file is found (indicated in the “Content-Type” field of the HTTP response header), it is downloaded to the specified directory. To select only those files that will be used for the purpose of generating music, two strategies are followed. Firstly, the crawler has the ability to directly download the files whose links contain a certain word (i.e., name of the composer). For the rest of the found files, they are downloaded and their metadata are analysed to look for information about the composer or the style if needed. If this information is not found, the file is automatically removed from the set of MIDI files used as the corpus.

For the crawler configuration, four fields have been included. The Target and Maximum Depth fields are required, as they are necessary for the crawling strategy. The words that are searched correspond to the labels of the problem *P*, in this way unrelated music files are avoided. The fourth field indicates where the MIDI files will be downloaded, and is initialized by default to a new folder in the user’s default folder. It is important to note that, before the training process begins, we might add new MIDI files manually, if we have some.

Each case *C* retrieved from the crawler or from the memory presents a solution *S* which contains a melody. This melody should be encoded in a standard format whether the music has been extracted from the Internet or extracted from the memory.

All the retrieved files need to be analyzed to extract the notes or pitches $N_{i}$ and the durations $D_{i}$. MIDI files contain different tracks that sound simultaneously. Each track represents a sequence of songs and there is a field that indicates what instrument it corresponds to. For the purposes of this work, only the melody is important in training the model. Therefore, the Composer Agent first looks for the field in which the type of instrument is encoded and selects only those tracks with a melodic instrument, discarding tracks that include drums or cymbals. Although initially we only have one MIDI file, it can be split into several files according to the number of melodic instruments that it contains.

The Composer Agent then analyzes each track to search for different events. Events of type NOTE_ON and NOTE_OFF [32] are particularly interesting for the purposes of this work, as they are used to determine the beginning or the end of a particular note. The next bytes that we can find in the MIDI file after such events gives the information about the pitches, silences or durations, useful for the current proposal. We should also mention the PROGRAM_CHANGE events that indicate the change of instrument and might be used in a future study to group the melodies according to the instrument the user wants to compose with.

The extraction of the pitches ($N_{i}$) is done automatically, as each musical note has its equivalent in MIDI number from 0 to 127. The extraction of the duration $D_{i}$ needs a more extensive work. Each file has information about the time signature and the mean velocity of each beat in seconds. With those numbers, we make a normalization of the onset time of each note event. Subtracting the onset time of two consecutive notes we obtain a value and then we make a discretization between 1 and 32, where 1 is a whole note, and 32 is a thirty-second note.

Subsequently, the melody extracted is encoded as a list of pairs <$N_{i},D_{i}$> and used as the training set for the Markov Models in the Reuse Stage.

### 5.2. Generating the New Melody (Reuse Stage)

**Figure 6:**
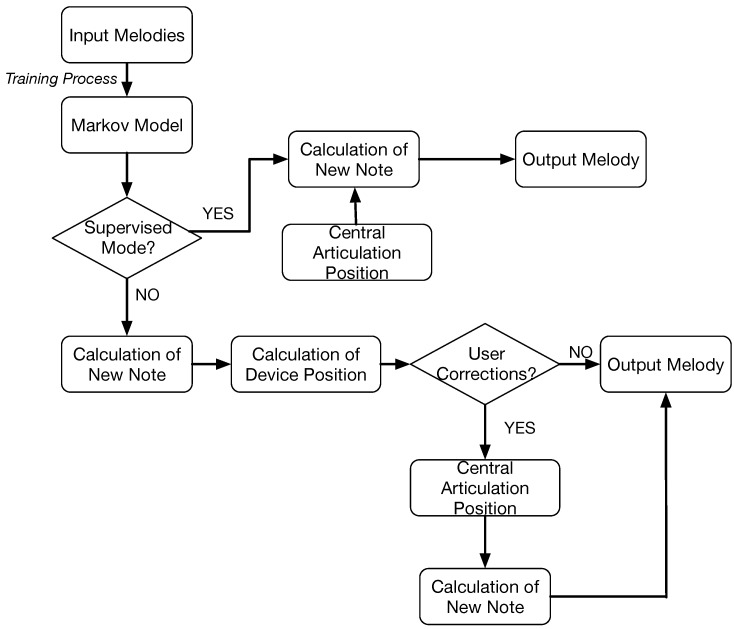
Schema with the adaptation process to generate a new melody.

The Composer Agent aims to generate a melody by applying not only the statistical model but also the interaction of the user with the mechanical device. Therefore, a hybrid approach is proposed and designed according to Figure 6.

Markov Models are a statistical tool that is widely used to model temporal properties in different fields, such as meteorology, economic fluctuations or market prediction. They are also applied to the automatic generation of music from a training set, also denominated “corpus”. The corpus contains information on music that helps to model a new musical composition and is representative of a particular style or author.

The Markov model is trained by iterating between all the sequences of the MIDI files, extracting the duration and pitch and progressively adding transitions to its corresponding state. Once the Markov Model is trained, it determines the most probable transition <$N_{i},D_{i}$>, where a transition means adding a new note according to the previous notes that have already been included in the melody. However, the transition that will be selected depends not only on the Markov Model, but also on the indications given by the user through the mechanical device.

When the Markov Model is ready, we set the control device to express both duration and pitch through the movement of the device’s arms along the 2D space (axes X and Y) as detailed in the previous section. It is important to highlight that each position of the central articulation of the mechanical device corresponds to a “reference” note (Y axis) and duration (X axis), also called “reference” transition $t_{r}$. Therefore, the same position can produce different notes in different moments, although they will be similar in duration and pitch. These different notes or transitions given by the device $t_{i}$ depend on the Markov Model that limits which notes are eligible for the melody.

After translating the position of the device into a reference note and duration $t_{r}$, the probabilities of the transitions $t_{i}$ should be calculated according to the Markov Model. The modification is influenced by the working modes of the system (Figure 6). In the supervised mode, the user controls the mechanical device without any external assistance from the machine. As the generated melody follows the users’ indications, the probabilities of the Markov Model also follow the users’ desires. On the contrary, in the collaborative mode the mechanical device can resist the users’ movements so that the melody is also partially controlled by the system. In this mode, the machine suggests new movements learned from previous experiences through the mechanical device. Consequently, the modification of probabilities in the Markov model depends on the decision of the system.

The probability of transitions that are close to the control rise as the function of parameter *k* increases, whose value oscillates between 0 and 1, depending on the working mode and determines the weight of the reference transition and of each transition $t_{i}$ available in the device (Equation (4)):

(4) $$P\left(t_{i}\right)=k\cdot P_{D}\left(t_{i},t_{r}\right)+\left(1-k\right)\cdot P_{M}\left(t_{i}\right)$$

where $P_{D}\left(t_{i},t_{r}\right)$ means the probability of the transition according to the device, and $P_{M}\left(t_{i}\right)$ represents the probability of a transition according to the Markov model. If the user chooses the supervisor mode, *k* is empirically set to 0.55.

In the supervised mode, the first step is to select some of the most probable transitions $t_{i}$ according to the Markov Model. The probability of a transition being selected according to the position of the device $P_{D}\left(t_{i}\right)$ is calculated from the distance between each possible transition $t_{i}$ and the reference transition $t_{r}$.

Each transition *t* consists of a pitch and a duration, thus, we can calculate two different distances, the distance to the note and the distance to the duration. The probability of $t_{i}$ to be selected depends on the euclidean distance $d_{p}$ between the reference pitch and the pitch of $t_{i}$, and the distance $d_{d}$ between the reference duration and the duration of the transition $t_{i}$.

(5) $$P_{D}\left(t_{i},t_{r}\right)=\left\{\begin{array}{cc} 1-||0.5\cdot d_{p}\left(t_{i},t_{r}\right)+0.5\cdot d_{d}\left(t_{i},t_{r}\right)\left|\right| & :d_{d}<d_{dmax}|d_{p}<d_{pmax}\\ 0 & :otherwise \end{array}\right.$$

The distance $d_{p}$ is calculated by applying the Euclidean distance of the *x*-position of $t_{r}$ and the *x*-position of $t_{i}$ following the device search space. The distance $d_{d}$ is also measured with the Euclidean distance of the *y* coordinate of $t_{r}$ and $t_{i}$. According to Equation (5) the likelihood of $t_{i}$ being selected is inversely proportional to the total distance between $t_{i}$ and the reference transition $t_{r}$.

The Markov Model sometimes provides some likely transitions but too far from $t_{r}$. To discard such cases, two adjustable parameters are set to determine the maximum distance of the notes in pitch $d_{pmax}$ and duration $d_{dmax}$ between transitions $t_{i}$ and $t_{r}$. When the duration or the pitch distances are above these thresholds, the probability $P_{D}$ is automatically set to 0, to avoid transitions that are too far from the control of the device. $MAX_{DD}$ and $MAX_{DP}$ values are empirically set to 4.3 and 6.2, respectively. Following the Markov Model, it can occur that all the possible transitions are beyond the control range. In such cases, the transition of the Markov model with the minimum distance between $t_{i}$ and $t_{r}$ is selected as the next note in the melody.

In the collaborative mode, *k* is set to 0, which means that the probability of a note being selected only depends on the Markov Model ($P_{M}\left(t_{i}\right)$). However, in order to preserve some coherent results and avoid some abrupt differences that could damage the device, the notes that can be considered as options should appear within the values of $d_{dmax}$ and $d_{pmax}$ where the center is the present position of the central articulation. That means the Composer Agent searches for the most probable notes and durations (transition $t_{i}$) to be selected as part of the melody that are comprised in the proposed radius. However, the automatic movement can be partially corrected by the user by resisting the force. This correction is translated into a new note, calculated by the Markov Model following the equations given in the supervised mode (Figure 6).

The user decides to finish the process and the set of notes can then be re-played for user evaluation, this is fully explained in the next section.

### 5.3. Validating the Final Melody (Review Stage)

The final musical result from the human-machine interaction should be rated to measure the level of user satisfaction. The obtained creative product depends on the user’s performance and their preferences, thus it is essential to validate the melody on the basis of their opinion. Consequently, the Composer Agent orders the Interface Agent to present the melody to the user and collect their opinion. Clearly, the melody should be evaluated by the user according to whose preferences it was composed, since the same melody may not be suited to the musical preferences of another user. The Interface Agent plays the created melody to the user; the player allows the user to reproduce the melody several times. The user is then asked to rate the melody according the degree of their satisfaction with the final result, meaning the degree to which the melody has been adapted to the indications they gave with the mechanical device. User satisfaction is represented on a categorical scale from 0 (totally unsatisfied) to 10 (totally satisfied). User opinion is sent to the Composer Agent, who decides whether the melody should be stored (making use of the Storage Agent).

### 5.4. Storing a New Case (Retain Stage)

The Composer Agent, considers the rating of the user and determines if the new melody (case) has enough quality to be stored for future use. Henceforth, a dynamic threshold is established, which depends on previously stored cases. For instance, if a user consistently gives a low rating to the generated melodies, a case with a global punctuation of 6 can be considered very high. On the contrary, in situations where cases generally receive high ratings, a score of 6 would be considered a low value. The threshold is established by the Storage Agent, who calculates the mean of all the global ratings of the compositions. If there are not enough data in the memory, the threshold is established at 4.

We consider this value to be representative of the lack of important musical compositions but it determines a starting point at which music is generated based on user experience. Likewise, we could analyze as a future work, a visualization of the melody generated online, in order to obtain ratings from other users and study the social acceptance of the music created with the system.

These melodies are incorporated into the memory of the system as a new case. Therefore, we store the *P* description (the initial labels selected by the user), the generated melody and the final rating that the user gave. In a future iteration, if the labels that the user provides coincide with the labels of this case, the melody is incorporated in the corpus for the markov training. This is done in order to adapt the learning model to the specific preferences of the user, as the melody has been partially generated by the device, although it respected the stylistic rules given by the Markov Models.

It is important to note that the melodies incorporated do not replace the external MIDI files added by the crawler. Additionally, in order not to “forget” the original musical style defined by the labels, there is a maximum number of user-generated melodies that can be used. This number represents the 30% of the total number of melodies used as the training corpus, and was set empirically.

## 6. Experimental Setting

Our proposal was deployed as a desktop application connected to the mechanical device. In order to preserve the different styles and preferences of the user when composing the melodies, each user has a profile with the different melodies generated by them. Each time the user creates a new melody they can select a general musical style or a specific composer the music must be based on.

For this experiment, we give a list of authors and styles for the users. For each option, a set of 100 files have been uploaded beforehand in order to speed up the process of file search, as the users could ask for some authors or styles where MIDI files were not found. The users can select the music of the styles: “Baroque”, “Classical”, “Romanticism”, “Jazz”, “Pop-rock”. For the composers, we suggest: “Vivaldi”, “Bach”, “Haydn”, “Mozart”, “Beethoven”, “Schubert”, “Albéniz”, “Louis Armstrong”, “Nina Simone”, “The Beatles” and “Bruno Mars” as possibilities. This list can be extended in a future study.

When the Composer Agent starts to create music, the user can guide the generated melody through the mechanical device (Device Agent) in the supervised mode, or the machine can guide the user through the force generated in the device following the Markov Model in the collaborative mode. It is important to note that the device is not intended to select a specific note to the generated melody, it is the user that provides an orientative guideline of pitch and duration to adapt the creation to a certain context.

**Figure 7:**
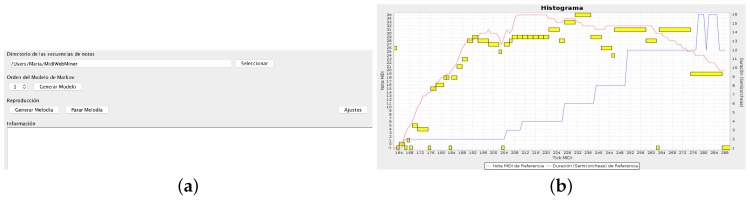
(**a**) Capture of the screen while the user is composing a melodic line; (**b**) Caption of the main interface.

To show this issue, the transitions between notes are registered and represented on the interface in real time, as shown in Figure 7. In the plot, the red line represents the pitch changes, while the blue line represents the rhythm changes experienced by the device. Yellow boxes are the final notes of the last melody played by the system, according to the Markov Models and the position of the central articulation. When the user finishes the creative process, the melody can be re-played or downloaded in MIDI format. Additionally, the Interface Agent presents the user with a brief questionnaire that evaluates his/her satisfaction with the musical result. Once the user answers, the process can be started again.

## 7. Experimental Setting

The evaluation of the system is threefold. Firstly, the system should learn from user preferences in order to improve its musical results. Therefore, a comparative study is performed to show the evolution of the overall ratings of different melodies when the number of executions gradually increases with a CBR agent or without it. Secondly, the system should make it possible for users to generate their own melodies. Therefore, a questionnaire is given to users to collect their opinions on the efficacy of the system in generating melodies that are suited to user preferences. Finally, interaction between humans and machines should be correctly implemented in order to successfully generate melodies in both supervised and collaborative modes. Therefore, two kinds of experiments were carried out, one analyzed the success of the two modes and the other the level of interaction between the user and the machine. Principally, the MAS-based design provides the system with a flexibility that can easily be extended, and the CBR architecture equips the system with a functionality that allows to compose different kinds of melodies and to add a learning component based on the opinions of the community.

The users were volunteers from different fields. In particular, we involved 9 users with some musical training and 28 users without any musical training. They have been selected to test the system during one month. A mean of 250 melodies per user were created and the MAS was implemented in order to learn about the users’ intentions according to the possible context. Some of the melodies can be accessed through our website: https://ethnomusicusal.wordpress.com.

Although the system can be used in different places, for the users’ convenience, over the month the experiments were conducted in the lab in which the system was initially installed. Before the test, they had two, ten minute sessions of rehearsal so that they could understand the work flow of the system and become familiar with the device. Before the users played with the system, they were told about how to manage the device and how to create music. They were recommended to think about the general structure of the music and how to generate the musical phrases with the device.

Over one month, they did between 13 and 17 sessions, where each session took between 15 and 20 min. The number of melodies generated in each session depended on the users, as they could create melodies of different lengths and selected those one that they liked to be stored in the memory. However, the mean of melodies generated at each session was usually about 15 short melodies.

The first part of the evaluation aimed to analyze whether the melodies generated in the final iterations of the system were considered by the users to be better than those generated in the initial executions of the application. For this purpose, in 10 sessions, some melodies were created with the CBR architecture, while others were generated without applying the learning architecture. This fact was not made known to the users during the tests.

After one month of testing, the users were familiar enough with the system to evaluate it as a whole. To validate the usefulness of the tool to generate adapted melodies , they were asked to answer a questionnaire in which four items are evaluated: the experience in using the whole system, the interface, the quality of the system to control the melody, and the adaptation of the system to the initial preferences of the user. Finally, we asked for an overall rating of the whole system.

The system is mainly thought for potential users with or without musical training that look for music adapted to their preferences and situations (i.e., they aim to create quiet music to help children fall asleep, to create specific music for therapy, etc.). Therefore, it is very important to know whether the users feel comfortable when they make use of the system to generate music. For this purpose, the interface should be easy to interpret and reflect the changes in the system.

As an interactive tool, the device is also essential for collaboration between the system and the users. Additionally, the device must faithfully reflect the intentions of the users through their movement. Consequently, we need to evaluate whether the users feel the device captures their preferences.

The system is also thought as a means to guide the generation of a melody according to the parameters established by the user. For example, if the user needs quite music that follows classical standards in a concrete context, the system should adapt to their preferences instead of creating a general melody. Therefore, the degree to which the system adapts is essential as it allows to satisfy the users’ needs (preferences, mood, etc.). Consequently, the users were also asked for the degree to which the system adapts. That means they should evaluate if the outcome respects the initial labels (style and/or author) they selected to generate the melody.

The third part of the test aimed to measure if the Device Agent collected the real movements of the device in the supervised mode and whether it translated them into a nice melody according to the style and user preferences. In this case, each user that participated in the experiments dedicated two of the sessions over the entire testing period, to test the system and generate at least 20 melodies for three different values of *k*.

## 8. Results and Discussion

This section presents the results of the three experiments. The first one intends to validate the learning architecture of the process. The second one aims to validate the features of the system. Therefore, a comparative study is presented with other recent works that had been conceived for similar purposes. This comparison helps to highlight the advantages of the present system. Additionally, a usability test and a listening test were also performed to evaluate the musical results of the system.

To demonstrate statistically the reliability of all experiments, we followed [33] theory to measure the statistical conditions of our listening test and user validation. As we stated before, the sample for the test contained 37 users with and without musical training. With this sample, we established the confidence level for the statistical conclusions, at the standard for most statistical analysis, 95%. The standard error, which is related to the distribution and the deviation of the parameters measured in the test, was set to 0.08654. The relative standard error for the listening test was 16.10%. This means the claims we can make according to the statistical results are under a maximum error of 16.10%, which is considered admissible according to [33].

### 8.1. Validating the Learning Process

**Figure 8:**
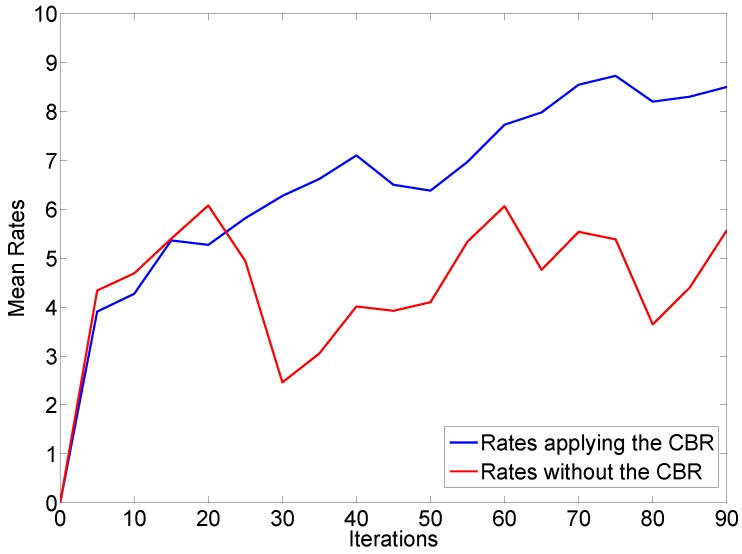
Evaluation of results in two different situations, with and without the CBR agent.

Firstly, we aimed to analyze if the melodies generated in the final iterations of the system were considered as better melodies than those generated in the initial executions of the application. Therefore, a comparative study was conducted between the MAS and Composer agents which implemented the CBR cycle and the agents that did not execute CBR. In the former situation the system learns from previous melodies, while in the latter situation, the system cannot learn from previous cases to improve the results. The users’ opinions were collected at every five system iterations, the results are presented in Figure 8, where the X axis represents the number of generated melodies and the Y axis shows the mean subjective ratings of the melodies.

The blue line represents the overall ratings when the CBR agent is integrated in the system. As we can see, the graph shows a general increase when the number of iterations raises, which demonstrates that the system learns from user preferences to improve the results. On the other hand, the red line plots the overall ratings when the CBR agent is not present in the system.

In the figure, we can observe that the red graph does not show any evolution. This is because the users ratings only depended on the ability of the Markov Model to generate a melody in a concrete execution . That means the CBR architecture is an essential tool which allows the system to learn from the users’ indications and improve the future results but respecting the tonal standards given by the initial parameters.

### 8.2. Evaluating the Performance of the System

In order to underscore the advantages of our system, we compared it with two other systems that generate music thorough collaboration between human and machine, namely The Continuator [12] and VirtualBand [15]. As we stated in Section 2, both systems share multiple features, what permits us to narrow down our focus to the generation process. A qualitative comparison is shown in Table 1.

In order to generate chords, all the analyzed proposals make use of a previous corpus of work. The three proposed systems are capable of assisting the user in the generation of melodies adapted to their personal experience. VirtualBand and The Continuator are designed to interact with musical instruments. Therefore, a musician is needed, whereas our system only involves a simple device which can be easily used by any user. Additionally, the device can guide the user in a concrete moment to show them possible transitions that can improve their knowledge of the musical style, unlike the rest of the analyzed tools.

Secondly, and in order to validate the efficacy of the system for the users, we asked them about their experiences after one month of testing. Firstly, we evaluated the usability of the system according to the users’ experience. We made use of the System Usability Scale. The System Usability Scale (SUS) is a simple, ten-item scale giving a global view of subjective assessments of usability [34]. The items evaluated are the following:

1. I think that I would like to use this system frequently
2. I found the system unnecessarily complex
3. I thought the system was easy to use
4. I think that I would need the support of a technical person to be able to use this system
5. I found the various functions in this system were well integrated
6. I thought there was too much inconsistency in this system
7. I would imagine that most people would learn to use this system very quickly
8. I found the system very cumbersome to use
9. I felt very confident using the system
10. I needed to learn a lot of things before I could get going with this system

Each item was evaluated with a Likert-scale from 1 (“Completely disagree”) to 5 (“Completely agree”). To calculate the SUS score, first sum the score contributions from each item. Each item’s score contribution can range from 1 to 5. For items 1, 3, 5, 7, and 9 the score contribution is the scale position minus 1. For items 2, 4, 6, 8 and 10, the contribution is 5 minus the scale position. Multiply the sum of the scores by 2.5 to obtain the overall value of SU.

We also aim to measure the satisfaction with the musical features such as the device and the adaptation of the music to the selected style. One of the key features of the music is related to the emotions and the believability of the music, meaning that the music might have been produced by humans. Therefore, we added six additional questions that were:

- In your opinion, does the outcome respect the initial labels (style and/or author) selected beforehand to generate the melody?
- Does the device capture the users’ intentions to control pitch and rhythm?
- Does the system allow to generate a nice melody that the user would not be able to compose without the use of the device?
- Does the system present some unexpected options at any point that enriches the melody? These are the transitions that contain properties which do not entirely comply with the stylistic standards but helps to preserve originality.
- Overall, does the system present melodies that, in some way, sounds convincing enough to imitate a human intention or style?
- Do you have any suggestion for the system improvement?

The users rated the previous items from 1 (“Completely disagree”) to 5 (“Completely agree”), except the final question, which is a text field to give some suggestion if they had any. For such categorical data, we use the chi-square test to prove that the results reflect the quality of the melodies according to the subjective ratings given by the listeners. It is important to note that we can get very subjective ratings since all listeners can have different interpretations of the musical pieces and different musical tastes. Thus, we considered the analysis of the median $M_{e}$ and the mode $M_{o}$ a useful step. Table 2 shows the results of the statistical analysis.

For all the items measured, the *p*-value obtained indicates that the null hypothesis should be accepted. The data are well-adjusted to a normal distribution, which is the expected theoretical distribution, which means the sample has been correctly selected.

The overall satisfaction rating of the usability is quite high, as the majority of the users evaluated the usability with a rating of 79. Additionally, the median shows that at least half of the users evaluated the system positively, suggesting that the users are mainly satisfied with their interaction with the system in terms of usability.

The system not only provides a pleasant melody, but also a melody that follows the style selected by the user. This parameter was rated highly by more than half the users, as the median and mode shows (4.09), showing that they liked this particular feature of the system. This indicates the users are satisfied about how the labels are considered by the system to generate a new melody that follows the standards given by the labels, although the dispersion shows some discrepancies.

The table shows that the degree of satisfaction with the system is quite high, with a median of 4. The users consider the system helps them to retrieve good and different melodies, what means that the chords have good musical quality according to the style context. Finally, the users are moderately satisfied with the amount of unexpected transitions that can retrieve (3), this means the system may create surprising motifs at some point of the progression. However, this feature can be improved in a future work by adding different parameters to the objective function, such as expectedness or tonal tension. Finally, the users consider that most of melodies are generated with quality enough to reflect some behavior properly human according to the melody line, with a median of 3. However, this feature can be improved, according to the mode 3, which means that most users evaluated the results as “fair human”.

In the evaluation questionnaire, some users suggested to add a complete stave to represent the generated melody instead of a melodic line, which means the visualization of data can be improved.

We cannot forget the evaluations showed are quite subjective, as they are subject to the users’ perception, feelings, culture and even mood. Therefore, it can exist some discrepancies, which are shown through the standard deviation for each item evaluated.

### 8.3. Analysis of the Operation Modes

The final part of the test aimed to measure if the Device Agent can collect the real movements of the device in the supervised mode and whether it translates them into a nice melody according to the style and user preferences. In this case, each participant dedicated two of sessions over the experimentation period to make use of the system when the constant *k* was very low (0.15) and then increased up to 0.55 and 0.85. They tested the system and generated at least 20 melodies for each value of *k*. Afterwards, we selected the 5 melodies with the highest ratings and a total of 15 were presented to each user to evaluate which ones they thought best represented their own style that they chose in the first stage of the system. The users can rate the melodies on a scale from 1 (not representing their style at all) to 5 (the melody completely represents their style). We expect that melodies with a k=0.55 are selected as the best ones. The results are shown in Table 3.

The table shows the median and mode ratings given by the users for the different *k* values analyzed. As we can see, the highest values correspond to the value 0.55 for *k* for the mode and median. When *k* is very high, the Markov models are almost ignored and the movement of the device is completely free, leading to a random melody that only follows the users indications but without any stylistic rule. Henceforth, users gave a low rating to the generated melodies due to their low musical quality. On the contrary, when *k* is very low, the melodies ignored the indications of users through the central articulation of the device. Thus, the users feel their preferences are not considered and therefore rate these melodies poorly.

To demonstrate the dependency of the three samples in this experiment, we performed the test of Kruskal Wallis, whose null hypothesis tries to demonstrate that the distribution are equal in the sense that none of the group populations is dominant over any of the others. The value of the K value and *p*-value indicates that the null hypothesis is accepted, meaning that the population are equally distributed. Therefore, the results are not overlapping and can be acceped statistically as independent.

However, although the participants liked the melodies guided by them over those auto-generated, that does not mean the melodies were better in terms of musical quality. The system is proposed to adapt to the users’ indications but also respect the tonal standards given by a style selected beforehand. Therefore, the users are asked to evaluate if the system balances these two aspects correctly in the melodies generated by it.

We performed a new experiment where a total of 25 melodies were created twice. The first time, 15 users who did not have any musical training were selected randomly. At the first session, they were told to follow the movements of the machine in the composition process. Then, the users repeated the same process but they were allowed to make some corrections in the melody whenever they found it appropriate. Finally, the users were asked to choose the melody that they preferred. From the total number of participants, 82.3% of users selected the melodies to which they had contributed. On the basis of the results, we can state that collaboration is required to obtain a more satisfactory product.

It is important to consider the fact that the objective of the system is to make the collaboration between the user and the AI fruitful. Both have complementary skills that contribute to the quality of the generated melody. On the one hand, the Composer Agent suggests plausible notes that comply with the rest of the sequence, the musical features of the style or the composer indicated by the labels. This is essential as it would be very hard for the users to create a pleasant piece of music that would follow the tonal standards of the user’s preferences (music which follows Bach’s style or jazz style). As a result, the Composer Agent is crucial as it achieves a “nice” melody through the Markov Models which follow musical constraints.

On the other hand, Markov models have no sense of global structure or evolution. Therefore, users can define phrases or a hierarchical structure, which are essential in every music style. The user can also adapt the creation to a certain context or situation. In this regard, the device allows the users to have some global control over the main structure. As a result of the “collaboration” that occurred in the creative process, the melody encompasses both aspects. Although the users are encouraged to plan their movement of the device beforehand, they often wish to improvise. This is an issue we should study more in-depth, as it leads us to wonder whether the melody can wake such intentions due to their particular features, or if it is only a way to toy with an interactive tool.

Although in the guided mode the user is deprived of an input device that would control the construction of the melody and therefore, the composer agent is deprived of the guidelines to create a long-term structure, we aimed to experiment with this mode to analyze the degree of adaptation of the melody generation, once several melodies generated by the user have been incorporated in the memory.

Finally, we have to note that the ratings are subjective scores, thus the results might differ from some users to others. However, we can conclude that the system can make melodic compositions that adapt to user preferences, mood or context in which the system is used. Among the improvements that could be added to this proposal in the future work, is the ability of the user to freely choose the author and the style of the composition and also to improve the interface.

## 9. Conclusions

In this work, a system was developed for the generation of music through mechanical device. Interaction is achieved in two parallel ways. On the one hand, the device collects user preferences in order to adapt the generated melodies to their musical tastes. On the other hand, the designed mechanical device can adapt to the system with two different modes. In the supervised mode, the user can direct the construction of the melody by moving this device. In the collaborative mode, the user can let the force produced in the control of the device to guide their movements. Consequently, a MAS was developed with a CBR agent (called Composer Agent), which equips the system with the ability to learn from previous experiences, improving the system’s performance.

Specifically, the device collects the input data needed to generate a melody and shows the output data, such as the melody. The Crawler and the Storage Agents successfully retrieve previous solutions or external MIDI files that coincide with the preferences of users displayed on the interface. With this information, the Composer Agent trains a Markov Model which is used to calculate the probability of a note being selected as the next in the sequence of notes that create the melody. In the supervised mode, the selection of notes is also influenced by the device, while in the collaborative mode, the Markov Model allows the system for a force expressed through the device to guide the user’s movements. Users are asked to rate the final melody generated, according to their general degree of satisfaction. This evaluation is then used to decide whether the musical solution (case) should be stored in the memory through the Storage Agent, or be removed because of its low quality.

The evaluation is aimed at analyzing the usefulness of the CBR agent in the system, the ability of the overall application to generate good musical results and encourage collaboration between humans and machines in both modes. The results indicate a high degree of satisfaction when the system integrates the preferences of the user in the generation of melodies. The results of the system with the implemented CBR agent tend to improve over time, when the number of executions increases constantly. Additionally, the overall ratings show that the device agent captures user and machine preferences very-well (depending on the mode) through the mechanical device. Likewise, the results are positive in relation to the ease of use of the system and are helpful in generating music adapted to personal preferences.

It is important to note that the device is essential to promoting collaboration between a human and a machine in the generation of music. However, it is designed to only capture pitch notes and durations. That means the Markov Model is only trained with notes and their duration, omitting other important features such as musical phrases or harmony. Likewise, the work is more focused on creating music that adapts the style according to the Markov Models than on expressing concrete emotions. However, emotions are one of the key features of music. We should analyze how to introduce this information through the device or other sensors to generate more interesting music or add a harmonization phase. Additionally, a more complex process for the retrieval of music could be implemented in order to include musical styles and not to depend on tagged MIDI files.

To improve the interaction of the users, we would like to analyze the incorporation of more standard devices, such as the mouse or a standard joystick, to indicate a more accurate feature related to pitch and rhythm duration. On the other hand, to improve the automatic generation of music, it is necessary to analyze other properties of the melodies, including a more general view of the composition. In general, the addition of the long-term music structure in the AI part signifies a deep study of harmony, tonal tension, hierarchical relationships between the music, as well as the incorporation of new mechanisms such as parallel Markov Models or hybrid learning models (ANN or bioinspired models mixed with Markov Models), which will be addressed in a future work. 

## Figures and Tables

**Table 1:** Comparison between our proposal and other proposals.

	Our Proposal	The Continuator	Virtual Band
Melody Generation	X	X	X
Music Theory Encoding	X	X	X
Phrase Construction	X	X	-
Interaction with the Users	X	X	X
Non-Musician Users	X	-	-
Device with Feedback	X	-	-

**Table 2:** Shows the final statistics when the users finished testing the system.

	Usability	Style Quality	Adaptation Degree	Music Quality	Unexpectedness	Humanness
$\chi^{2}$ (*p*-value)	9.8704 × 10${}^{-8}$	0.013436327	0.001393571	0.010634032	8.18924 × 10${}^{-5}$	−
$M_{e}$	71	4	4	4	4	3
$M_{o}$	79	4	4	4	4	3

**Table 3:** Shows the final statistics when the users finished testing the system.

	k=0.15	k=0.55	k=0.85
Kruskal Wallis (K)		0.02642389	
Kruskal Wallis (*p*-value)		0.02642389	
Median	4	4	3
Mode	3	4	3
